# Are Cervical Pessaries Effective in Preventing Preterm Birth?

**DOI:** 10.7759/cureus.51775

**Published:** 2024-01-06

**Authors:** Morgan Goodell, Leilani Leechalad, Varun Soti

**Affiliations:** 1 Obstetrics and Gynecology, Lake Erie College of Osteopathic Medicine, Elmira, USA; 2 Internal Medicine, Lake Erie College of Osteopathic Medicine, Elmira, USA; 3 Pharmacology and Therapeutics, Lake Erie College of Osteopathic Medicine, Elmira, USA

**Keywords:** cervical pessary, multiple pregnancy, preterm birth, progesterone, singleton pregnancy

## Abstract

Preterm births are a significant concern, as they can have serious consequences for both infants and mothers. It is crucial to identify risk factors associated with preterm birth and to implement effective interventions, such as progesterone, cervical pessary, and cervical cerclage, to prevent it. This systematic review aims to evaluate the efficacy of cervical pessary in reducing spontaneous preterm delivery. However, cervical pessaries have limited research and conflicting findings when compared to other interventions for preventing preterm labor. Therefore, this review seeks to analyze various studies to evaluate their overall effectiveness and better understand their role in treating this vulnerable population. The literature search was conducted using PubMed between January and September 2023, and the systematic review adhered to the Preferred Reporting Items for Systematic Reviews and Meta-Analyses (PRISMA) guidelines. The results of this review highlight the importance of continued research into mitigation strategies for preterm birth. There is some evidence that indicates that using cervical pessaries before 34 weeks can be effective. While some studies have reported positive outcomes when cervical pessaries are combined with other treatments like progesterone, there is no solid statistical evidence to support this claim. Furthermore, additional research is needed to comprehend the impact of singleton pregnancies and long-term outcomes for both mothers and infants.

## Introduction and background

Approximately 15 million infants are born prematurely every year [1]. In 2021, the United States saw around one out of 10 infants born prematurely [2]. Preterm birth significantly contributes to morbidity and mortality in newborns, especially in multi-pregnancies [3]. It refers to delivery before 37 weeks of gestation. However, the causes of most preterm births remain unknown, and they can be influenced by multiple factors. The short-term and long-term complications associated with prematurity have a profound impact on families worldwide. Prematurity increases the risk of neonatal intensive care unit stays, neonatal sepsis, necrotizing enterocolitis, cerebral palsy, and chronic lung disease in infants [4]. Moreover, preterm birth is associated with long-term complications such as neurodevelopmental delays, lower cognitive and motor skills, and academic performance compared to term-born children. It also increases the likelihood of attention deficit/hyperactivity disorder and behavioral issues [5]. Additionally, mothers of preterm babies are at an increased risk of experiencing anxiety, depression, post-traumatic stress disorder, and detachment from their newborns [6].

These complications impact the entire family, both emotionally and financially. Identifying risk factors for preterm birth is crucial, although many cases remain unexplained. Advanced maternal age is often considered a risk factor for women older than 35. Smoking and higher body mass index also contribute to the risk of preterm delivery [7]. Intrauterine infection from a vaginal or urinary infection can also predispose women to preterm birth [8]. Two strong predictors of preterm birth are shortened cervical length and fetal fibronectin, particularly in women with multiple pregnancies. Cervical length is measured via transvaginal ultrasound between 16 and 24 weeks of gestation. A cervix is considered short if its length is ≤25 mm [9]. The shorter the cervix, the higher the risk of preterm birth. Fetal fibronectin levels can also help assess the risk of preterm birth. Fibronectin, a glycoprotein in the amniotic membranes, can be found in vaginal and cervical secretions. Recent studies indicate that high fibronectin levels increase the risk of preterm birth [10].

The risk of premature birth can be assessed by measuring cervical length and fibronectin levels. However, detecting and managing these risk factors early for effective prevention is crucial. Previous research has demonstrated a delay in preterm birth by implementing early interventions to extend gestational age. Notably, interventions such as progesterone, cervical pessary, and cervical cerclage are commonly employed to safeguard against preterm birth. Progesterone, a vital hormone in females, governs the menstrual cycle and pregnancy. Its role encompasses facilitating follicular development, corpus luteum secretion, and creating an optimal environment for successful embryo implantation. Such secretions persist until the placenta assumes the responsibility of progesterone production to support fetal growth [11].

Progesterone is pivotal in regulating trophoblast migration, reducing uterine contractions throughout pregnancy, suppressing the maternal immune system to prevent immune response against the embryo, and preparing mammary tissue for breastfeeding [11]. A decline in progesterone levels is thought to contribute to cervical ripening leading up to birth [12]. Extensive investigations have studied progesterone therapies for years to prevent preterm birth, with the administration of vaginal progesterone demonstrating promising results in reducing preterm birth before 34 weeks of gestation [13].

Cervical pessaries have been utilized to address bladder incontinence, prolapse, and preterm birth [13]. When fitted to the cervix with small diameters and fixed against the pelvic floor with larger diameters, these silicone rings effectively rotate the cervix posteriorly to correct the cervical angle and reduce the risk of preterm birth. The procedure of cervical cerclage, on the other hand, involves temporarily closing the cervix with sutures during the second trimester of pregnancy (13 to 26 weeks) in cases of cervical insufficiency [14]. Cervical insufficiency refers to the dilation and shortening of the cervix, often painless and progressively dilated in the second or third trimester. This condition results in microbial invasion of the amniotic cavity, membrane prolapse, spontaneous rupture of membranes, or preterm birth [15]. Consequently, suturing the open cervical os can decrease the risk of preterm birth by preventing infection and spontaneous membrane rupture that may lead to microbial invasion of the intrauterine cavity. It is worth mentioning that despite its potential benefits, cerclage comes with certain risks. These risks include higher rates of perinatal morbidity and mortality, as well as occurrences of maternal fever, bleeding, and discharge [16].

Despite the interventions mentioned above, some patients and obstetricians opt for a “wait for birth” approach known as expectant management [17]. Studies on the management of preterm birth have yet to yield conclusive results on effective treatment strategies. In addition, the use of cervical pessaries as a preventive method for preterm birth has limited research and conflicting findings when compared to other ways, such as vaginal progesterone alone or progesterone-eluting pessaries. There is a notable gap in the literature when comparing pessaries to other treatment options and determining their effectiveness in preventing preterm labor. Therefore, we conducted this systematic review to analyze various studies and evaluate the efficacy of cervical pessary use in reducing spontaneous preterm delivery. Our aim is not only to assess the overall effectiveness of these interventions but also to foster a better understanding of their role in treating this vulnerable population.

## Review

Method

Literature Search and Study Selection

We conducted a literature search following the guidelines of Preferred Reporting Items for Systematic Reviews and Meta-Analyses (PRISMA) [18]. We searched PubMed and utilized specific keywords to optimize our search results. These included “cervical pessary preterm birth,” which yielded 263 results. Another keyword, “cervical pessary for preventing preterm birth,” resulted in 223 matches. “Cervical pessary with progesterone” gave us 155 results, while “pessary, progesterone, preterm birth” produced 159.

We also used “pessary, singleton,” and “pessary twins,” which gave us 145 and 42 results, respectively. Additionally, we used “cervical pessary” and “vaginal progesterone,” which generated 570 and 5,521 results, respectively. Only relevant clinical studies in English were included in this review.

**Figure 1:**
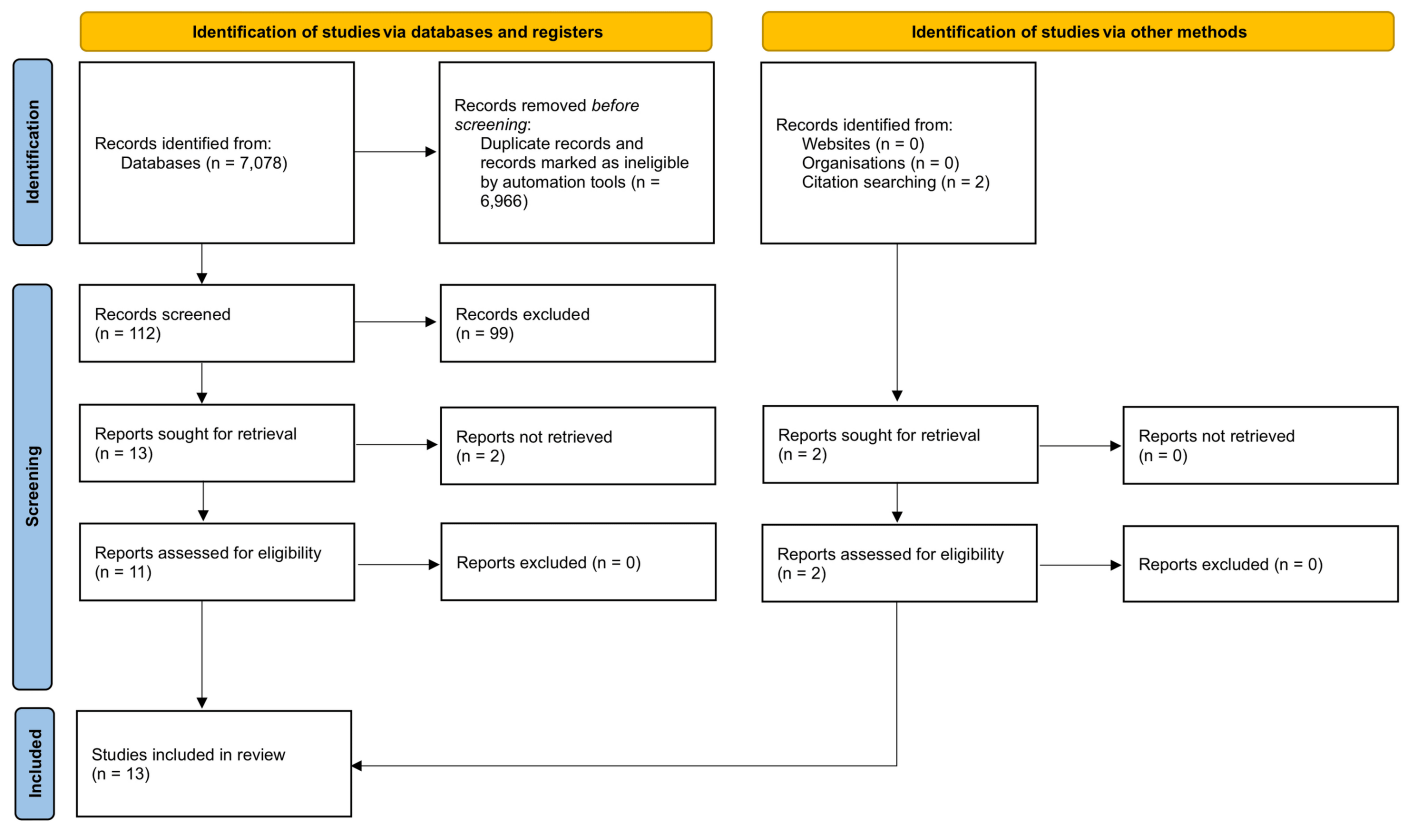
Literature search and study selection process This review utilized PubMed to search for literature between January 2023 and September 2023 that met inclusion criteria, including RCTs and articles on combined treatment of preterm birth. The search was narrowed down to 13 studies. n, number; RCTs, randomized control trials

Figure 1 shows the PRISMA flowchart illustrating the methodology. We included clinical articles that met the criteria of being randomized control trials (RCTs), open-label studies, meta-analyses, or economic analyses. We also considered studies that used a cervical pessary and/or progesterone during either twin or singleton pregnancy, regardless of a preterm birth history. Our literature search focused on studies published in the last 15 years. Based on prior literature, we assigned a level of clinical evidence to the selected studies [19].

Please refer to Table 1 for this review's inclusion/exclusion criteria.

**Table 1:** Study selection criteria In this review, we included studies published in English within the last 15 years, focusing on treatment strategies for preterm birth and meeting our inclusion criteria. RCTs, randomized control trials

Inclusion criteria	Exclusion criteria
RCTs	Prospective cohorts
RCTs	Retrospective cohorts
Open-label studies	Case reports
Single-blind studies	Pre-clinical studies
Double-blind studies	Narrative reviews
Studies on singleton pregnancies	Commentaries
Studies on twin pregnancies	
Studies focusing on cervical pessary alone	
Studies focusing on cervical pessary in combination with progesterone	
Studies focusing on progesterone alone	
Metanalysis of RCTs	

Management of Preterm Pregnancies

The researchers in the literature (included in this review) examined various common strategies to mitigate preterm birth. These included pessaries, progesterone, combinations of progesterone and pessaries, and expectant management. Expectant management or standard care served as the control in multiple articles. The control comprised follow-up visits every two to four weeks, with progesterone doses of 100 mg, 200 mg, or 400 mg, administered until 33.6-36.6 weeks of pregnancy. Patients also underwent ultrasounds and vaginal swabs to detect infections. Progesterone was administered either independently or in combination with pessaries. Dosages included 100 mg, 200 mg, and 400 mg and could be delivered as vaginal suppositories or drug-eluting pessaries. Cervical pessaries were used either as a standalone treatment or in conjunction with progesterone. A practicing physician inserted the cervical pessaries and removed them between 34 and 36.6 weeks. Combination treatment assessed the efficacy of combining pessary and progesterone in reducing preterm birth risk. The combination of pessary and progesterone treatments ranged from 100 mg to 400 mg. These treatments constituted the main groups investigated in these studies.

Results

The results are documented in Table 2 highlighting the key findings of the studies reviewed in this paper.

**Table 2:** Key studies highlight the effectiveness of cervical pessaries in preventing preterm birth The table presents key studies that evaluate the efficacy of cervical pessaries in managing preterm delivery and compare their effectiveness with expectant management, vaginal progesterone alone, or a combination of progesterone and pessary. PTB, preterm birth; p-value, probability variable; RCT, randomized control trials

Authors	Study design	Level of evidence	Sample size	Treatment method	Parameters assessed	Study findings
Saccone et al. (2017) [20]	RCT	I	300 women	Cervical pessary vs. expectant management	Spontaneous PTB less than 34 weeks	Supported pessary use. Significantly lowered risk for spontaneous PTB (p=0.04)
Nicolaides et al. (2016) [21]	RCT	I	1180	Cervical pessary vs. expectant management	Spontaneous PTB less than 34 weeks	No support of pessary use (p=0.722)
Nicolaides et al (2016) [22]	RCT	I	935 girls and women	Cervical pessary vs. expectant management	Spontaneous PTB less than 34 weeks	No support of pessary use (p=0.57)
Goya et al. (2016) [23]	RCT	I	137	Cervical pessary vs. expectant management	Spontaneous PTB less than 34 weeks	Supported pessary use. Significantly decreased PTB (p=0.03)
Groussolles et al. (2022) [24]	RCT	I	315	Cervical pessary vs. expectant management	Adverse neonatal outcomes	No support of pessary use (p=0.320)
Norman et al. (2021) [25]	RCT	I	503	Cervical pessary vs. expectant management	Spontaneous PTB less than 34 weeks	No support of pessary use (p=0.54)
Shabaan et al. (2018) [26]	RCT	I	140 women	Cervical pessary plus progesterone vs. expectant management	Spontaneous PTB less than 37 weeks	No support of combination progesterone pessary use (p=0.06)
Barinov et al. (2020) [27]	RCT	I	217	Cervical pessary plus progesterone vs. progesterone alone	Improved placental migration in placenta previa	Supported combination progesterone pessary (p=0.037)
Crowther et al. (2017) [28]	RCT	I	740 women	Cervical pessary plus progesterone vs. pessary alone	Incidence of neonatal respiratory distress and severe respiratory distress syndrome	No support of combination progesterone pessary use (p=0.798)
Dang et al. (2019) [29]	RCT	I	300 women	Cervical pessary vs. progesterone alone	Spontaneous PTB less than 34 weeks	No support of pessary over progesterone use for primary outcome (p=0.24)
Zhuang et al (2023) [30]	Systemic review and meta-analysis	II.2	16 studies (five were RCT)	Cervical pessary plus progesterone vs. progesterone alone	Spontaneous PTB less than 37 weeks	No support of combination progesterone pessary (p=0.82)
Karbasian et al (2016) [31]	RCT	I	144 women	Cervical pessary plus progesterone vs. progesterone alone	Spontaneous PTB less than 37 weeks	No support of combination progesterone pessary (p=0.36)

Cervical Pessary Alone Versus Expectant Management

Cervical pessaries are devices used to prevent uterine prolapse, bladder leakage, and preterm birth. In a study conducted by Saccone et al. (2017), the effectiveness of cervical pessary use during singleton pregnancies was evaluated in reducing the risk of spontaneous preterm labor in women with a short cervical length and no prior history of preterm birth. An RCT was conducted on 300 singleton pregnant women with short cervical length and no previous history of spontaneous preterm birth (SPB), who were randomly assigned to either receive a cervical pessary or no cervical pessary (control group). Both groups of women with cervical measurements less than 20 mm were given 200 mg progesterone until their 36th week and six days of pregnancy. The study found that the use of cervical pessary resulted in a lower risk of SPB, with only 7.3% of patients having spontaneous labor in the pessary group compared to 15.3% in the non-pessary control group (p=0.04). These results suggest that cervical pessaries may be an effective intervention in reducing the risk of preterm birth in women with a short cervix and no prior history of preterm birth [20].

Goya et al. (2016) conducted a multicenter study to investigate the efficacy of cervical pessaries in reducing preterm birth in twin pregnancies with ultrasound-determined short cervix. The study enrolled 137 women with sonographic cervical lengths less than 25 mm, who were randomly assigned to receive cervical pessary or expectant management in a 1:1 ratio. The primary outcome was SPB at less than 34 weeks gestation. The study found that the incidence of SPB less than 34 weeks was significantly lower in the pessary group (16.8%) compared to the expectant management group (39.4%) (p=0.03). This study provides evidence that cervical pessaries could be an effective intervention to reduce the risk of preterm birth in twin pregnancies with a short cervix [23].

The studies conducted by Saccone et al. (2017) and Goya et al. (2016) have shown that the use of cervical pessaries for both singleton and twin pregnancies can significantly reduce the risk of preterm birth in patients with short cervical length. These findings are in contrast to the results of Nicolaides et al. (2016) and Nicolaides et al. (2016), which did not demonstrate any significant difference in preterm birth rates between patients treated with cervical pessaries and those who were not, in either singleton or twin pregnancies [21,22].

Nicolaides et al. (2016) enrolled 932 pregnant women with singleton pregnancies and a sonographic cervical length of less than 25 mm between 20 and 24.6 weeks in an RCT. The participants were randomly assigned to receive either a cervical pessary or expectant management. The study aimed to investigate the differences in the rate of spontaneous delivery before 34 weeks gestation between the two groups. The results showed no significant differences in the rate of SPB before 34 weeks of pregnancy between the pessary group and the expectant management group. Specifically, 55 women in the pessary group and 50 in the expectant management group experienced SPB before 34 weeks of gestation, with a p-value of 0.57 [21].

Nicolaides et al. (2016) conducted a multicenter, RCT to evaluate the effectiveness of cervical pessary in twin pregnancies during the second trimester in preventing SPB before 34 weeks of gestation. The study involved 1,180 women randomly assigned to two groups: cervical pessary (590 women) and expectant management (590 women). This trial showed no significant difference in the incidence of preterm birth between the two groups. The rate of preterm birth in the cervical pessary group was 13.6%, while in the expectant management group, it was 12.9% (p=0.722) [22].

Groussolles et al. (2022) conducted an open-label, multicenter, RCT to investigate the efficacy of the Arabin cervical pessary in reducing adverse neonatal outcomes in twin pregnancies with a short cervix. The study enrolled 315 twin pregnancies with cervical length measuring less than 35 mm, with random allocation to either the pessary placement group or the standard care group at 16-24 weeks gestation. The study aimed to determine if the use of the Arabin cervical pessary was associated with a reduction in preterm birth rates among twin pregnancies with a short cervix [24].

The study’s findings demonstrated no significant difference in the preterm birth rates between the two groups (p=0.32). This implies that the Arabin cervical pessary did not have a substantial effect on reducing adverse neonatal outcomes in twin pregnancies with a short cervix. These results suggest that further studies may be necessary to determine the efficacy of the Arabin cervical pessary in reducing the risk of preterm birth in twin pregnancies with a short cervix [24].

Norman et al. (2021) conducted an RCT on 503 women with twin pregnancies with a cervical measurement of less than 35 mm across multiple centers. The study aimed to compare the efficacy of cervical pessary with standard care (250 women) versus standard care alone (253 women). The results showed that cervical pessary had little to no effect on reducing preterm birth (18.4% versus 20.6% for expectant management; p=0.54) [25].

The incidence of twin pregnancies is on the rise globally, primarily due to assisted reproductive techniques and maternal age at first pregnancy. Preterm delivery is a significant complication in such cases, and preventive treatments like progesterone or cervical cerclage have not been studied adequately. Although cervical pessaries are often suggested as a preventative measure, their effectiveness in preventing preterm birth and related morbidity and mortality is debatable.

Expectant Management Versus Combination

Progesterone is a crucial component of the female reproductive cycle and plays a vital role in maintaining pregnancy. Extensive research has been conducted on progesterone's efficacy in managing short cervical length and preventing preterm birth. While progesterone administration remains a standard treatment in pregnancy, combining it with a cervical pessary may offer potential benefits, such as prolonging gestational age and mitigating the risk of preterm birth. Shabaan et al. (2018) conducted a randomized, open-label, controlled trial at three different centers in Egypt from February 2015 to January 2017 to assess the efficacy of vaginal progesterone in preventing preterm delivery in twin pregnancies and its effect on perinatal outcomes. The study included 70 women who were randomly assigned to two groups. Group 1 received a 400 mg dose of progesterone via vaginal pessary administered every night from week 28 until delivery, while group 2 received standard prenatal care and served as the control. Notably, no significant differences were observed between the groups concerning delivery before 37 weeks (p=0.06) or the mode of delivery (vaginal versus c-section; p=0.31). Thus, the researchers concluded that vaginal administration of 400 mg progesterone doses via pessary after 28 weeks of gestation did not prevent preterm birth in twin pregnancies [26,27].

Crowther et al. (2017) conducted a multicenter RCT spanning six years to evaluate the efficacy of vaginal progesterone pessaries in reducing the risk and severity of respiratory distress syndrome in newborns, as well as to compare the rate of preterm birth in women receiving vaginal progesterone pessaries to those receiving expectant management. The study involved 787 women with live singleton or twin pregnancies between 18 and 23.6 weeks of gestation and with a previous history of preterm birth associated with cervical incompetence or preterm rupture of membranes. Participants were randomized into two groups: group 1, consisting of 398 women who received 100 mg of progesterone pessary, and group 2, consisting of 389 women who received a non-drug-eluting pessary. Both the researchers and participants were masked to study treatment, and the women were instructed to use their treatment [28].

The findings of the study indicated that the percentage of patients in the progesterone group (36.5%) and the control group (37.2%) who were born before 37 weeks was not significantly different (p=0.765). Therefore, the study did not support the use of vaginal progesterone pessaries in women with a history of preterm birth to reduce the risk of neonatal respiratory distress or other neonatal and maternal morbidities associated with preterm birth [28].

Cervical Pessary Versus Progesterone

In an RCT conducted by Dang et al. (2019), the efficacy of cervical pessary and vaginal progesterone was compared for preventing preterm birth in women with twin pregnancies and short cervixes (defined as less than 38 mm). The study included 300 asymptomatic women with twin pregnancies and short cervical lengths who were randomly assigned to two groups. Group 1 consisted of 150 women who received the Arabin Pessary, while group 2 consisted of 150 women who received once daily 400 mg vaginal progesterone [29].

The primary outcome of the study was preterm birth before 34 weeks of gestation. Preterm birth before 34 weeks occurred in 16% of the pessary group and 22% of the progesterone group (p=0.24). However, in women with cervical lengths of 28 mm or less, pessary use significantly reduced the preterm birth rate from 46% to 21% (p=0.47) and improved the composite of poor perinatal outcomes (p=0.38). Overall, the study found that both cervical pessary and 400 mg vaginal progesterone resulted in similar rates of preterm birth before 34 weeks gestation in women with twin pregnancies and cervical length less than 38 mm. However, for cases with births before 24 weeks, pessary use notably reduced the preterm birth rate [29].

Cervical Pessary With Progesterone Versus Progesterone Alone

In their study, Barinov et al. (2020) investigated the efficacy of progesterone-only treatment versus progesterone treatment combined with cervical pessary use for managing placenta previa and typical cervical lengths. An RCT was conducted, which included 217 women at high risk for preterm labor who received progesterone treatment at seven weeks of pregnancy. At weeks 18-20, patients were randomly assigned to two groups: group 1 continued receiving progesterone-only treatment, while group 2 underwent cervical pessary insertion. The patients were monitored regularly, and ultrasound examinations assessed placenta migration. The study revealed that the combination treatment led to a statistically significant decrease in preterm births under 34 weeks compared to progesterone-only treatment, with rates of 8.6% and 23.5%, respectively (p=0.031). This finding emphasizes the potential benefits of using a combination of progesterone and cervical pessary for the management of placenta previa and typical cervical lengths in women at high risk for preterm labor [27].

The study showed that patients with a cervical pessary had more favorable changes in their anterior cervical-uterine angle and arcuate artery resistive index than those with progesterone treatment. Furthermore, the patients with a pessary had 1.8 times more movement in placental migration. These findings suggest that a cervical pessary may be used in placenta previa activity, preventing preterm birth in these patients. However, further research is required to validate the findings, generalize the data to a larger population, and understand the complete mechanism of its use in placenta previa [27].

In contrast, Zhuang and colleagues (2023) conducted a meta-analysis that evaluated the efficacy of cervical pessary utilization in conjunction with progesterone to prevent preterm birth. The study reviewed 16 articles, including 10 singleton studies, of which five were RCTs and six were cohort studies. Additionally, two cohort studies were focused on twin pregnancies. The results indicated that although vaginal progesterone was found to significantly reduce preterm birth less than 34 weeks, as revealed by the cohort meta-analysis, the use of vaginal progesterone versus combination therapy (cervical pessary + progesterone) in the RCT did not demonstrate a notable reduction in preterm birth for any gestation weeks less than or equal to 37 weeks of gestation (p=0.82). The findings suggest a discrepancy in the efficacy of combination therapy and progesterone in reducing preterm birth. The researchers recommend conducting more extensive studies to determine its effectiveness accurately. The current body of literature on the efficacy of cervical pessary in reducing preterm birth is limited, and the existing studies have revealed conflicting outcomes. Therefore, the researchers utilized this study as a benchmark to determine the efficacy of reducing SPB in women with singleton pregnancies and short cervical length in the second trimester [30].

Karbasian et al. (2016) conducted a multicenter RCT to evaluate the efficacy of cervical pessary combined with vaginal progesterone in comparison with vaginal progesterone alone in reducing the risk of spontaneous preterm delivery. The study enrolled 144 singleton pregnant women with short cervical length, determined by ultrasound measurement of less than or equal to 25 mm. The participants were equally divided into two groups, with 73 participants in each group. The study assessed the patients monthly after the initiation of the intervention. The study showed that the combination group did not have a significantly different rate of spontaneous preterm delivery compared to the group receiving progesterone-only (p=0.36) [31]. 

Discussion

Neonatal morbidity and mortality are frequently caused by prematurity, which is characterized as delivery before 37 weeks of gestation. This can have adverse effects on both the newborn and the mother. It can lead to various complications in the short-term and long-term, such as low birth weight, respiratory problems, cerebral palsy, underdeveloped brains, and an increased risk of mortality. According to Hirvonen et al. (2014), the incidence of cerebral palsy was observed to be higher in preterm infants compared to term infants. Maternal complications of preterm birth include chorioamnionitis, endometritis, trauma, and increased risk for preterm birth in future pregnancies [32].

Several risk factors have been identified in women for preterm birth, including multiple gestation, assisted reproduction in the current pregnancy, and shortened cervical length. Preterm birth significantly increased in multiple gestations, especially twin gestations, with 31.2 twins per 1,000 births reported in the United States in 2021 [33]. Cervical length has been shown to help predict preterm birth in singleton and twin pregnancies, particularly when paired with gestational age at measurement. Shorter cervical lengths have been associated with higher preterm birth rates [34].

Multiple gestation is a significant risk factor for preterm birth, with more than half of the 13 studies reviewed focusing on twin gestations. Crowther et al. (2017) was the only study to include singleton and twin pregnancies in their study groups, but they did not separate the groups by gestation. It is crucial to examine the optimal mitigation method to avoid morbidity and mortality in twin infants, as twin gestations are associated with a higher risk of preterm birth [28].

Extensive research has been dedicated to various measures to prevent premature birth. Cervical pessaries, vaginal progesterone, cervical cerclage, and progesterone-eluting pessaries have been explored as strategies. However, conflicting findings have been observed. Some studies support the use of pessaries, while others argue against it. In a systematic review, researchers found no significant difference in the use of combination progesterone pessaries compared to a placebo, progesterone alone, or expectant management. Studies on cervical pessaries without progesterone also yielded conflicting results. Some papers demonstrated statistical significance for their primary outcomes, while others showed no statistical significance. It is essential to note that this comparative study has limitations, as no studies focused on cervical cerclage or progesterone-only therapy compared to expectant management.

The primary outcomes of these studies mainly focused on the length of gestational age at delivery. Most studies listed preterm birth before 37 weeks as a primary outcome, while a few examined fetal and maternal complications. For instance, Barinov et al. (2020) explored the effects of cervical pessary and progesterone combination on maternal placenta previa resolution compared to progesterone-only treatment [27]. A group of researchers led by Crowther (2017) carried out a study to find ways to reduce the chances of respiratory distress syndrome in babies born to women who had given birth prematurely in the past. Their aim was to make sure that these newborns were healthy and did not face any breathing difficulties. They used vaginal progesterone pessaries as a means to achieve this objective [28]. The PESSARONE trial conducted by Groussolles et al. (2022) investigated the potential reduction of adverse neonatal effects in twin pregnancies with short cervixes using the Arabin pessary [24].

Despite having different primary outcomes, the reviewed studies shared similar secondary results with numerous overlaps. The secondary outcomes for neonates included birth weight, APGAR scores, neonatal death, neonatal intensive care unit admission and length of stay, sepsis, respiratory distress syndrome, and necrotizing enterocolitis. On the other hand, maternal secondary outcomes included vaginal discharge, gestational age at the time of delivery, gestational age at trial start, pain during removal or insertion of the pessary, and infection. Of the reviewed studies, Barinov et al. (2020) observed a significant rise in placenta previa resolution using combination progesterone pessaries compared to progesterone-only [27].

Additionally, they observed a decrease in SPB in their study group. Nicolaides et al. (2016), however, observed no significant difference in SPB in singleton pregnancies with pessary use versus expectant management. They did, however, observe a statistically significant increase in vaginal discharge in women who used pessaries [22]. Dang et al. (2019) reported a substantial decrease in neonatal composite poor perinatal outcomes with cervical pessary use [29]. According to an economic analysis of an RCT conducted by Le et al. (2020), cervical pessaries are a practical and cost-effective option for preventing preterm birth in twin gestations. The study concluded that this treatment is easily accessible and could be a viable solution for expectant mothers. The findings of this study suggest that cervical pessaries can serve as a viable alternative to more invasive and expensive treatment options. This study underscores the importance of exploring affordable and accessible means of treatment for preterm birth, particularly in twin gestations, where the risk of preterm birth is significantly higher [35]. Similarly, Goya et al. (2016) found cervical pessaries to be beneficial in preventing preterm birth in twins, citing the method as an affordable and safe treatment [23].

## Conclusions

Preventing preterm birth is of utmost importance, and this review emphasizes the need for ongoing research into effective strategies. One approach that has been effectively explored is the use of cervical pessaries before 34 weeks of pregnancy. While some studies have reported positive results when combining cervical pessaries with other treatments like progesterone, there is no solid statistical evidence to support this claim. Additionally, more research is needed to understand the impact of singleton pregnancies and long-term outcomes for both mothers and infants. This avenue of investigation could provide valuable insights into the potential links between premature birth and adult health issues.
