# Correction: Are Cervical Pessaries Effective in Preventing Preterm Birth?

**DOI:** 10.7759/cureus.c258

**Published:** 2025-08-26

**Authors:** Morgan Goodell, Leilani Leechalad, Varun Soti

**Affiliations:** 1 Obstetrics and Gynecology, Lake Erie College of Osteopathic Medicine, Elmira, USA; 2 Internal Medicine, Lake Erie College of Osteopathic Medicine, Elmira, USA; 3 Pharmacology and Therapeutics, Lake Erie College of Osteopathic Medicine, Elmira, USA

This article has been corrected to fix the following typo and formatting issue in the PRISMA diagram:

"Records removed before screening: Duplicate records and records marked as ineligible by automation tools" has been corrected from 0 to 6,996 and reformatted for consistency with PRISMA design.

